# Supplementary material for: Empirical methods for controlling false positives and estimating confidence in ChIP-Seq peaks
Source: BMC Bioinformatics. 2008 Dec 5;9:523. doi: 10.1186/1471-2105-9-523 (PMC2628906; doi:10.1186/1471-2105-9-523)
Supplement: Additional File 1 — A variety of html documents from the USeq web site detailing the available applications, their best usage, output file type descriptions, command line menus, etc. [file 1471-2105-9-523-S1.zip › USeqUserGuides/JavaDoc.zip!/JavaDoc/bioroot/LoginBean.html]

LoginBean


|  |  |  |
| --- | --- | --- |
| **Overview** \| **Package** \| **Class** \| **Use** \| **Tree** \| **Deprecated** \| **Index** \| **Help** | |  |
| **PREV CLASS**   **NEXT CLASS** | **FRAMES**    **NO FRAMES**     **All Classes** |  |
| SUMMARY: NESTED \| FIELD \| CONSTR \| METHOD | DETAIL: FIELD \| CONSTR \| METHOD |  |


---


## bioroot Class LoginBean

```
java.lang.Object
  bioroot.LoginBean
```

---

``` public class LoginBean extends java.lang.Object ```

**Author:**
:   nix
    Bean to hold and check login data

---

**Constructor Summary**

`LoginBean()`

`LoginBean(java.lang.String labGroupName,
java.lang.String loginName)`


| **Method Summary** | |
| --- | --- |
| `boolean` | `checkParams(DBUtil bioRoot)` |
| `UserBean` | `createUserBean(DBUtil bioRoot, java.lang.String host, java.lang.String IP)` |
| `java.lang.String` | `getChallenge()` |
| `java.lang.String` | `getChallengeDate()` |
| `java.lang.String` | `getDemoResponse()` |
| `java.lang.String` | `getLabGroup()` |
| `int` | `getLabGroupId()` |
| `java.lang.String` | `getLoginName()` |
| `java.lang.String` | `getMessages()` |
| `java.lang.String` | `getPassword()` |
| `java.lang.String` | `getResponse()` |
| `java.lang.String` | `getType()` |
| `UserBean` | `getUserBean()` |
| `int` | `getUserId()` |
| `boolean` | `isMakeChallenge()` |
| `boolean` | `paramsPresent()` |
| `void` | `setChallenge(java.lang.String string)` |
| `void` | `setChallengeDate(java.lang.String string)` |
| `void` | `setDemoResponse(java.lang.String demoResponse)` |
| `void` | `setLabGroup(java.lang.String string)` |
| `void` | `setLoginName(java.lang.String string)` |
| `void` | `setMakeChallenge(boolean b)` |
| `void` | `setMessages(java.lang.String string)` |
| `void` | `setPassword(java.lang.String string)` |
| `void` | `setResponse(java.lang.String string)` |

**Methods inherited from class java.lang.Object**

`equals, getClass, hashCode, notify, notifyAll, toString, wait, wait, wait`

**Constructor Detail**

### LoginBean

```
public LoginBean()
```

---


### LoginBean

```
public LoginBean(java.lang.String labGroupName,
                 java.lang.String loginName)
```


**Method Detail**

### paramsPresent

```
public boolean paramsPresent()
```

---


### checkParams

```
public boolean checkParams(DBUtil bioRoot)
```

---


### createUserBean

```
public UserBean createUserBean(DBUtil bioRoot,
                               java.lang.String host,
                               java.lang.String IP)
```

---


### getLoginName

```
public java.lang.String getLoginName()
```

---


### getPassword

```
public java.lang.String getPassword()
```

---


### setLoginName

```
public void setLoginName(java.lang.String string)
```

---


### setPassword

```
public void setPassword(java.lang.String string)
```

---


### getUserBean

```
public UserBean getUserBean()
```

---


### getMessages

```
public java.lang.String getMessages()
```

---


### setMessages

```
public void setMessages(java.lang.String string)
```

---


### getLabGroup

```
public java.lang.String getLabGroup()
```

---


### setLabGroup

```
public void setLabGroup(java.lang.String string)
```

---


### getChallenge

```
public java.lang.String getChallenge()
```

---


### getResponse

```
public java.lang.String getResponse()
```

---


### getType

```
public java.lang.String getType()
```

---


### getUserId

```
public int getUserId()
```

---


### setResponse

```
public void setResponse(java.lang.String string)
```

---


### getChallengeDate

```
public java.lang.String getChallengeDate()
```

---


### setChallengeDate

```
public void setChallengeDate(java.lang.String string)
```

---


### setChallenge

```
public void setChallenge(java.lang.String string)
```

---


### isMakeChallenge

```
public boolean isMakeChallenge()
```

---


### setMakeChallenge

```
public void setMakeChallenge(boolean b)
```

---


### getLabGroupId

```
public int getLabGroupId()
```

---


### getDemoResponse

```
public java.lang.String getDemoResponse()
```

---


### setDemoResponse

```
public void setDemoResponse(java.lang.String demoResponse)
```


---


|  |  |  |
| --- | --- | --- |
| **Overview** \| **Package** \| **Class** \| **Use** \| **Tree** \| **Deprecated** \| **Index** \| **Help** | |  |
| **PREV CLASS**   **NEXT CLASS** | **FRAMES**    **NO FRAMES**     **All Classes** |  |
| SUMMARY: NESTED \| FIELD \| CONSTR \| METHOD | DETAIL: FIELD \| CONSTR \| METHOD |  |


---
